# Response to letter regarding “Prospective randomized trial comparing relapse rates in dogs with steroid‐responsive meningitis‐arteritis treated with a 6‐week or 6‐month prednisolone protocol”

**DOI:** 10.1111/jvim.17189

**Published:** 2024-09-12

**Authors:** Jeremy H. Rose, Colin J. Driver, Lorna Arrol, Thomas J. A. Cardy, Joana Tabanez, Anna Tauro, Ricardo Fernandes, Imogen Schofield, Sophie Adamantos, Nicolas Granger, Thomas R. Harcourt‐Brown

**Affiliations:** ^1^ Lumbry Park Veterinary Specialists CVS Group PLC Alton UK; ^2^ Cave Veterinary Specialists Linnaeus Group Wellington UK; ^3^ College of Veterinary Medicine North Carolina State University Raleigh North Carolina USA; ^4^ Blaise Referrals IVC Evidensia Referrals Birmingham UK; ^5^ CVS Group PLC Diss UK; ^6^ Paragon Veterinary Referrals Linnaeus Group Wakefield UK; ^7^ Bristol Vet Specialists CVS Group PLC Bristol UK; ^8^ Langford Small Animal Hospital Bristol Veterinary School, University of Bristol Bristol UK

Dear Dr Woodward,

We thank you for your considered comments.

We believe we have been transparent in our methodology and the limitations of the study, particularly relating to the sample size and therefore certainty of the results.

We credit our readers with the ability to draw their own conclusions on the statistical and clinical relevance of the work based on the study design. We believe this prospective, randomized, multicenter trial offers real‐world findings that suggest a possibility that shorter prednisolone courses may be effective in treating SRMA, but agree this is not conclusive. As such, we used the word “could” in our statement, “both a ‘long’ 6‐month or ‘short’ 6‐week prednisolone protocol could be considered to treat SRMA cases.” It is possible neither prednisolone protocol used in our study is optimal because of the limited evidence on this topic to date. Hopefully this will be one of many studies to allow veterinary surgeons to optimize the medication and duration of the treatment to improve the quality of life for SRMA dogs.

We appreciate there may be a wider discussion concerning the utilization of *P*‐values in veterinary clinical research and appreciate your perspective on this matter. However, beyond noting this, we feel it is not for us to comment further.

Regards,

